# Oral Bioavailability Enhancement of Anti-Cancer Drugs Through Lipid Polymer Hybrid Nanoparticles

**DOI:** 10.3390/pharmaceutics17030381

**Published:** 2025-03-17

**Authors:** Saud Almawash

**Affiliations:** Department of Pharmaceutics, College of Pharmacy, Shaqra University, Shaqra 11961, Saudi Arabia; salmawash@su.edu.sa

**Keywords:** anti-cancer drug delivery, lipid polymer hybrid nanoparticles (LPHNs), oral chemotherapy, limited bioavailability, oral bioavailability enhancement

## Abstract

Cancer is considered as the second leading cause of death worldwide. Chemotherapy, radiotherapy, immunotherapy, and targeted drug delivery are the main treatment options for treating cancers. Chemotherapy drugs are either available for oral or parenteral use. Oral chemotherapy, also known as chemotherapy at home, is more likely to improve patient compliance and convenience. Oral anti-cancer drugs have bioavailability issues associated with lower aqueous solubility, first-pass metabolism, poor intestinal permeability and drug absorption, and degradation of the drug throughout its journey in the gastrointestinal tract. A highly developed carrier system known as lipid polymer hybrid nanoparticles (LPHNs) has been introduced. These nanocarriers enhance drug stability, solubility, and absorption, and reduce first-pass metabolism. Consequently, this will have a positive impact on oral bioavailability enhancement. This article provides an in-depth analysis of LPHNs as a novel drug delivery system for anti-cancer agents. It discusses an overview of the limited bioavailability of anti-cancer drugs, their reasons and consequences, LPHNs based anti-cancer drug delivery, conventional and modern preparation methods as well as their drug loading and entrapment efficiencies. In addition, this article also gives an insight into the mechanistic approach to oral bioavailability enhancement, potential applications in anti-cancer drug delivery, limitations, and future prospects of LPHNs in anti-cancer drug delivery.

## 1. Introduction

Cancer remains a leading cause of death worldwide, demanding continuous innovation in therapeutic strategies. Significant efforts have been made to advance cancer treatment modalities, particularly chemotherapy; however, challenges persist in achieving optimal therapeutic outcomes. Chemotherapy, a cornerstone of cancer treatment, can be administered through either parenteral or oral routes [1]. While parenteral administration ensures rapid systemic drug delivery, it often requires hospital visits, invasive procedures, and careful monitoring, which can impact patient compliance. On the other hand, oral chemotherapy, often referred to as “chemotherapy at home”, offers a more convenient alternative and potentially improves the quality of life for cancer patients [2]. In contrast to the present intravenous methods, oral delivery of anti-cancer drugs may improve patient compliance, safety, and convenience [3].

Numerous chemotherapy drugs, such as taxanes (e.g., paclitaxel and docetaxel), have poor oral bioavailability due to limited solubility, first-pass metabolism, and poor absorption in the gastrointestinal (GI) tract. For example, oral paclitaxel faces significant challenges with solubility and bioavailability, limiting its therapeutic potential [4,5]. Oral anti-cancer drugs often encounter physiological and pharmacokinetic barriers, including chemical instability, efflux transport, and first-pass metabolism, leading to reduced absorption and variability in pharmacokinetics and pharmacodynamics [6,7]. The desired therapeutic effect of anti-cancer drugs may be compromised if a significant amount is not absorbed from the gastrointestinal tract or if the drug is eliminated before reaching the tumor site, leading to reduced treatment effectiveness and suboptimal tumor control [8]. Additionally, drug resistance may arise as a result of the low availability of drug molecules for the target sites of the cancerous cells [9]. Variations in drug absorption among patients, influenced by factors such as genetic makeup, metabolic processes, and concurrent medications, can complicate the determination of optimal dosages, resulting in subpar therapeutic outcomes. Moreover, the high cost of anti-cancer medications, combined with low bioavailability, may require higher doses or more frequent administration, escalating treatment expenses and placing a significant burden on both patients and healthcare systems [10,11,12]. Therefore, choosing a suitable nanocarrier delivery system is of the utmost importance to overcome issues associated with conventional drug formulations and attain high-efficiency oral delivery.

Lipid-polymer hybrid nanoparticles (LPHNs) have emerged as a promising nanocarrier system to overcome these challenges, offering a versatile platform for improving oral bioavailability and optimizing cancer treatment outcomes [13]. LPHNs are core-shell nanoparticles composed of a lipid shell surrounding a polymer core [14]. The properties of polymeric nanoparticles and liposomes have been integrated to develop LPHNs. The LPHNs consist of a lipid coating encompassing a polymer core that is loaded with a drug [15]. This unique structure enables the extended circulation of the drug in the bloodstream and provides protection during its passage through the complex gastrointestinal environment. Additionally, the design of LPHNs effectively prevents water from accessing the drug-containing core [16,17,18]. Typically, they improve the stability of drugs in biological fluids, as well as versatile drug loading, controlled release capacity, high cellular absorption efficiency, desirable pharmacokinetics, and extended circulation half-life [19,20]. LPHNs have become a highly developed nanocarrier system that can overcome various challenges associated with oral anti-cancer drug delivery [21].

In order to solve the problems of loading water-soluble, ionic drugs in the hydrophobic solid lipid phase and achieving proper loading and sustained release of such drugs, LPHNs were first developed in the late 1990s and early 2000 [22]. Since then, a number of formulations have been designed to encapsulate a single anti-cancer drug in combination with a chemosensitizer, an anti-cancer drug with a P-glycoprotein inhibitor, or a combination of two anti-cancer drugs that work synergistically [23,24,25]. Additionally, the formulation of siRNA or siRNA paired with anti-cancer drugs has been developed for administering biological products such as gene therapy and immunotherapy. Promising findings from recent studies have demonstrated that LPHN-based anti-cancer drug delivery avoids efflux transporter-mediated multidrug resistance (MDR) in cancer cells and increases anti-tumor efficacy, enhances the bioavailability, while also decreasing the systemic toxicity of the anti-cancer drugs [26,27,28].

To date, multiple studies have attempted to report the outcomes of preclinical studies and lab experiments in the area of LPHN synthesis methods, composition, drug loading strategies, and therapeutic potentials [29,30,31]. However, the mechanistic explanations for the oral bioavailability enhancement of chemotherapy drugs through the utilization of LPHNs are not compiled in a single study. The present review aims to cover the explanation and state-of-the-art process through which LPHNs enhance the oral bioavailability of chemotherapy drugs.

## 2. Anti-Cancer Drugs with Limited Bioavailability Issues

### 2.1. Factors Influencing Oral Bioavailability of Anti-Cancer Drugs

The term “bioavailability” refers to a drug’s in vivo performance and defines how quickly and thoroughly it reaches systemic circulation, thus, making it available for therapeutic action [32]. Drug stability in the gastrointestinal tract, its solubility in water, rate of dissolution, intestinal epithelium permeability, stability against metabolic enzymes (intestinal and liver), and P-glycoprotein efflux pump are the main factors influencing the oral bioavailability of the drug [33,34]. Most anti-cancer drugs have bioavailability issues due to the P-glycoprotein efflux effect. Numerous chemotherapy drugs, such as camptothecin, topotecan, topoisomerase II inhibitors (etoposide and teniposide), doxorubicin, anthracyclines and vinca alkaloids, face issues including reduced bioavailability and interpatient variability [35].

Drug-metabolizing enzymes, drug efflux pumps and transporters are the main physiological variables affecting anti-cancer drugs’ oral bioavailability [36]. For example, a cytotoxic drug called etoposide is used for treating several kinds of cancers including small-cell lung cancer, different types of lymphomas, and germ-cell tumors. The oral bioavailability of etoposide is around 47–76% [37]. The oral bioavailability of etoposide is constrained by a number of variables, including enzyme metabolism (CYP3A4) and instability in the stomach and intestinal fluids. The oral bioavailability of topotecan, a Topoisomerase-I inhibitor, is only 40% [38]. Exemestane, an irreversible aromatase inhibitor, is primarily used as a first-line therapy for estrogen receptor-positive breast cancer patients. However, complex physicochemical characteristics limit its oral bioavailability (<10%) and anti-breast cancer efficacy [39]. Additionally, an antimetabolite called fluorouracil (5-FU) is used to treat a number of solid tumors. Even though oral and intravenous preparations of 5-FU exist, its oral absorption is lower and oral bioavailability is very unpredictable and variable [40]. Poor water solubility is a characteristic of small-molecule tyrosine kinase inhibitors, including pazopanib, vemurafenib, and lapatinib, which is a crucial factor for their limited absorption and bioavailability. The bioavailability of erlotinib, gefitinib, and pazopanib is negatively affected by antacid co-administration. In the case of taking tyrosine kinase inhibitors with antacids, the area under the curve decreases by 50% [41]. Ibrutinib has a remarkably poor bioavailability (2.7%), which contributes to the large interpatient variability in drug exposure with oral treatment [42]. In light of the aforementioned chemotherapeutic drugs with limited bioavailability, it becomes evident that an advanced drug delivery approach is imperative. The poor bioavailability exhibited by these drugs highlights the necessity for a more sophisticated method of administering them.

### 2.2. Planning Oral Formulation to Bypass Limited Bioavailability Issue

Complex formulation issues, such as decreased aqueous solubility, degradation by enzymes in the GI tract, first-pass metabolism of the drug by the liver cytochrome P450 (CYP3A4) and P-glycoprotein (intestine), and poor drug permeability through intestinal walls, should be addressed when planning oral delivery of such drug agents [43]. Pharmaceutical nanotechnology also known as chemotherapeutic engineering, cancer nanotechnology, or nanomedicine suggests that oral chemotherapy can be carried out by formulating the drugs in a variety of nanocarriers, such as LPHNs, solid lipid nanoparticles (SLNs), nanoemulsion and micelles, which are able to avoid P-gp recognition and non-specific cellular uptake of the loaded drug and facilitate prolonged, regulated, and targeted chemotherapy [44,45,46].

## 3. Lipid Polymer Hybrid Nanoparticles (LPHNs)

### 3.1. Basic Formulation Concept

**Figure 1:**
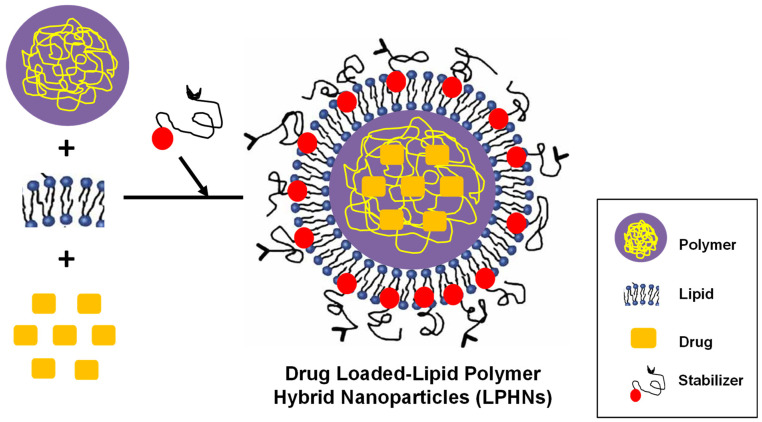
Composition of LPHNs. They are composed of three parts; (i) A monolayer of phospholipids around the core, (ii) A hydrophilic polymeric layer outside the lipid core, and (iii) A biodegradable hydrophobic polymeric core encapsulating drug agent.

The LPHNs are composed of three parts; a monolayer of phospholipids around the core to enhance overall biological compatibility, a hydrophilic polymeric layer outside the lipid to ensure formulation stability, and a biodegradable hydrophobic polymeric core to encapsulate hydrophobic drugs (as illustrated in Figure 1) [47,48]. The lipid shell helps to provide stability and appropriate biocompatibility, while the polymeric core is employed for carrying a variety of small molecules (drugs). Because of their outstanding biodegradability and biocompatibility, poly (lactic-co-glycolic acid) (PLGA) along with polycaprolactone (PCL) are the most utilized polymers. Lipids come in a variety of forms and can be utilized to create nanoparticles. Zwitterionic, cationic, anionic, and neutral phospholipids, such as lecithin, 1,2-dipalmitoyl-sn-glycero-3-phosphocholine or 1,2-dio-leoyl-sn-glycero-3-phosphoethanolamine, cholesterol, and myristic acid, as well as polyethylene glycol conjugate, are some of the frequently used lipids [49,50].

Several formulation processes have been reported and are used to produce LPHNs. These methods are based on the chemical and physical characteristics of the underlying components as well as the desired therapeutic outcome [51,52]. The ionic strength of the continuous phase, which is the aqueous medium containing the lipid vesicles and polymer NPs, the charge of the lipid formulation, and additional factors including size homogeneity of preformed lipid vesicles, the ratio of lipid vesicles to polymeric nanoparticles, all affect the physical properties such as size homogeneity and colloidal stability of LPHNs [53]. LPHNs may generally be synthesized using two different methods. In the two-step method, a polymer core and lipid shell are made separately first and subsequently combined at the end, while the alternative single-step method uses a one-step approach that involves single-step nanoprecipitation and self-assembly to create hybrid nanoparticles. The surface of the resultant LPHNs is often further functionalized with targeting ligands enabling payload distribution to specific cells or tissues for biomedical purposes (active targeting) [29,53].

### 3.2. Preparation Methods

#### 3.2.1. Single-Step Approach

**Figure 2:**
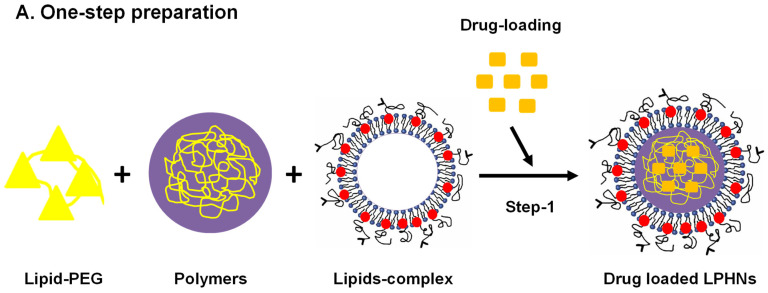

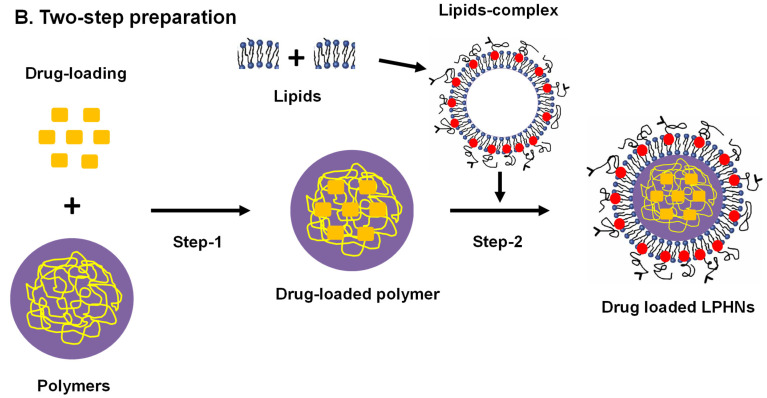
Schematic illustration of drug-loaded LPHN preparations, (**A**) Single-step (**B**) Two-step preparation. PEG: polyethylene glycol.

LPHNs with a lipid monolayer shell are frequently produced using the one-step method. In this technique, lipids and lipid PEG conjugates are dissolved in an aqueous solution whereas free polymers and hydrophobic drugs are dissolved in an organic solvent, and the used organic solvent must be a water-miscible solvent, such as acetonitrile. A small quantity of water-miscible organic solvent can be added to the aqueous solution to help with the solubilization of phospholipids in the solution. Following that, the gradual addition of the polymer solution is made to the lipid aqueous solution. Accelerated diffusion of the organic solvent into the aqueous solution allows the polymer to precipitate as nanoparticles. Through hydrophobic interactions, the lipids and lipid polyethylene glycol (PEG) will self-assemble on the surface of polymer nanoparticles (Figure 2A) [54,55].

The hydrophilic group of lipids extends into the surrounding aqueous environment, whereas the hydrophobic groups of lipids adhere to the hydrophobic polymer core. The lipid PEG conjugate is involved in the self-assembly process, as its PEG molecules are positioned on the outside while its lipid moiety is inserted inside the lipid monolayer for a stabilizing core. Lipids and lipid PEG conjugates may self-assemble at a temperature above the lipid phase transition. A hydrophobic polymer like PLGA should be utilized since the self-assembled lipid monolayer occurs because of hydrophobic interactions. This one-step self-assembly method is an inexpensive, feasible, and accurate method for producing LPHNs [56]. As an example, a recent study reported the preparation of folic acid decorated palbociclib loaded LPHNs using a single-step approach involving PLGA, with a lactic acid to glycolic ratio of (65:35)], and L-α phosphatidylcholine, respectively. The resulting LPHNs exhibited particle size of 143.36 ± 5.24 nm, 0.172 ± 0.004, zeta potential of −16.84 ± 0.27 mV, and % encapsulation efficiency of 93.12 ± 0.43, and an approximately 9, 11-fold reduction in IC_50_ values compared to free palbociclib in MCF-7 and MDA-MB-231 cells at 48 h [57].

#### 3.2.2. Classical Two-Step Approach

Most small-scale LPHN preparations are carried out utilizing conventional approaches. These procedures are usually employed in small-scale preparations for hybrid nanoparticles (Figure 2B). By using high-pressure homogenization, nanoprecipitation, or solvent evaporation during emulsification, polymeric nanoparticles can be developed. There are two types of conventional two-step methodologies. The thin lipid film can be dried by dissolving it inside an organic solvent like chloroform and evaporating it in a rotary evaporator or it can be transformed into preformed lipid vesicles by hydrating it. Differential centrifugation is employed in the purification process to separate free lipids and LPHNs [58,59].

#### 3.2.3. Modern Two-Step Approach

This novel approach is mostly used for developing a significant amount of LPHNs. The procedure includes mixing lipid vesicles with polymeric nanoparticles. Innovative techniques such as spray drying and soft lithography particle molding are used to create LPHNs. The 400–500 nm nanoparticles are produced by spray drying and then distributed in dichloromethane, an organic solvent that includes a wide range of lipid molecules. To make dry powdered LPHNs, lipid polymeric solutions are spray-dried using the processes of spray drying (SD) and spray freeze drying (SFD) [60,61].

Next, the organic solvent polymer, a sheet of Polyethylene terephthalate, is coated with PLGA that has been dissolved. Polyethylene terephthalate is heated in close contact with a soft lithography molding method, also known as particle replication in non-wetting templates (PRINT mold), which causes the polymer to flow into the mold and solidify when the temperature is lowered to room temperature. After that, LPHNs are created by removing nanoparticles from the mold and separating them from Polyethylene terephthalate sheets that had been coated with poly (vinyl alcohol) (PVA) using an aqueous lipid solution. After freeze-drying, these particles must exhibit a (+) 5 mV zeta potential and be needle-shaped and in 200 nm length [62,63].

#### 3.2.4. Self-Assembled Nanoprecipitation Method

**Figure 3:**
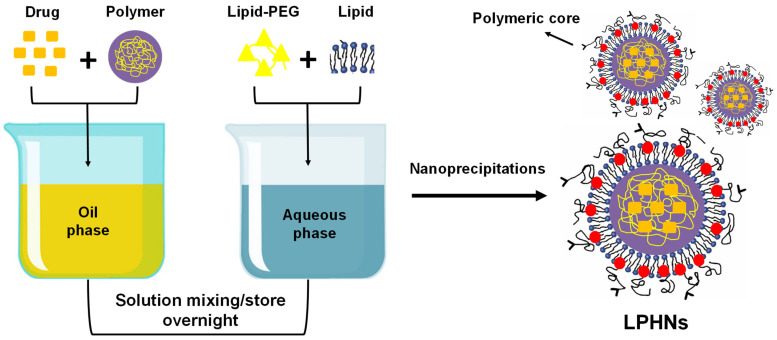
Preparation of LPHNs using the nanoprecipitation (self-assembled) method.

By employing this self-assembled nanoprecipitation technique, significant yields of LPHNs smaller than 100 nm may be achieved. The lipid shell and polymer core are made separately in two steps, and they are then combined to form a bilayer. A drug, polymer, and lipid are combined with each other in one phase during the self-assembly nanoprecipitation process to produce a monolayer of nanoparticles (Figure 3). LPHNs of dextran sulphate are prepared using a self-assembled nanoprecipitation approach to increase the efficacy of vincristine sulphate encapsulation and oral bioavailability [64,65].

#### 3.2.5. Single Emulsification Solvent Evaporation

**Figure 4:**
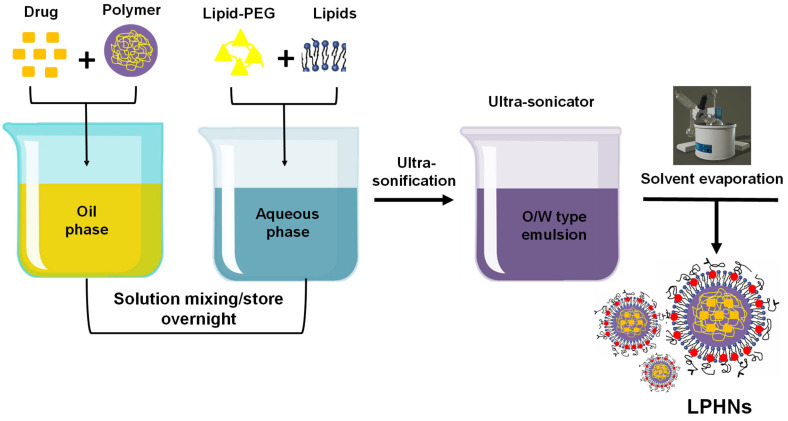
Preparation of LPHNs using the single-step emulsification solvent evaporation technique.

In this method, the polymer and drug to be encapsulated are dissolved in the organic (oil) phase. Using ultrasonic stirring, the solution is then gradually added to a lipid-water dispersion medium, forming an oil-in-water (o/w) emulsion. A rotary evaporator is employed to evaporate the organic solvent under reduced pressure, resulting in the formation of a polymer core, while the lipid-PEG conjugates self-assemble around the core (Figure 4). This technique is preferred over nanoprecipitation for the formulation of various LPHNs due to its ability to form stable emulsions. It is commonly used in the preparation of LPHNs loaded with nucleic acids for cancer treatment [18,66].

#### 3.2.6. Dual Emulsification Solvent Evaporation

**Figure 5:**
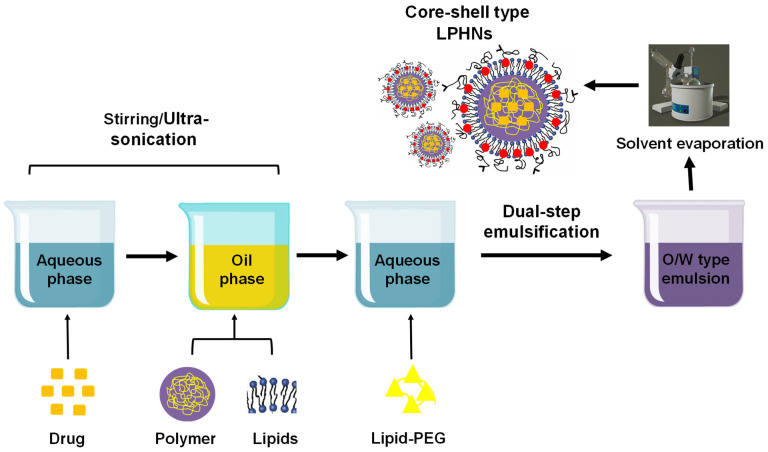
Preparation of LPHNs using the dual-step emulsification solvent evaporation technique.

The two-fold emulsification solvent evaporation procedure is the most efficient method for the production of polymeric nanoparticles. This method is essential to develop core-shell type LPHNs as well as polymeric nanoparticles. It provides a high encapsulation capability for both hydrophobic and hydrophilic drugs. This method is mostly used for determining drugs that are easily soluble in water but insoluble in the organic phase. The creation of (w/o/w) emulsion often employs this approach. A drug is first combined with an aqueous polymeric solution and an organic solvent that contains a lipid to form a (w/o) emulsion. A new (w/o/w) emulsion originates by shifting the emulsion to another aqueous medium. LPHNs are produced when the oil phase of an emulsion is evaporated using a rotary evaporator (Figure 5). This method is used to encapsulate nucleic acids and chemotherapy drugs [61,67].

### 3.3. Characterization and Assessment of % Drug Loading (DL) and % Encapsulation Efficiency (EE)

Characterization techniques are employed to evaluate various aspects of LPHNs, including their size, surface charge, morphology, drug loading, release kinetics, and biocompatibility. Methods like dynamic light scattering, electron microscopy, and spectroscopy are utilized to analyze particle size, shape, and surface properties. Drug loading and encapsulation efficiency can be assessed using chromatography or spectrophotometry. In vitro release studies examine controlled release patterns, while cell viability assays and in vivo studies investigate biocompatibility and toxicity. These characterization methods collectively provide valuable insights to optimize and comprehend the properties of LPHNs as effective drug delivery systems for chemotherapeutic drug agents [68].

In the context of drug delivery systems like LPHNs, the purpose of drug loading (DL) and encapsulation efficiency (EE) is to assess the effectiveness of these nanoparticles (NPs) in loading and delivering chemotherapeutic drugs. DL refers to the amount of drug that can be loaded into the NPs, while EE measures the efficiency of drug encapsulation within the NPs. The DL and EE of the NPs are important indexes for drug delivery systems. The DL and EE of LPHNs determined for paclitaxel were 27.71% and 92.24%. The high DL and EE values prove the effectiveness of the NPs of the lipid monolayer shell and polymeric core to load anti-cancer drugs [19].

Based on the data from Table 1, the LPHNs demonstrate a wide range of EE% for various anti-cancer drugs, making them versatile carriers for chemotherapeutic agents. Various researchers have attempted to formulate LPHNs using different lipid and polymer compositions. The EE% of these drugs varies depending on the type of lipid, polymer, and drug that was loaded, as well as the method of preparation. The preparation method with the highest encapsulation efficiency (EE%) is W/O/W double emulsification used in the preparation of curcumin loaded in a mPEG-PLGA copolymer [69], and 5-FU, irinotecan, oxaliplatin loaded in mPEG-PLA opolymer [70], which showed an EE% of 96% for both studies. Similarly, using dual-step sonication [28], single-step solvent evaporation [71], and single-step nanoprecipitation [72] also showed the highest EE% of 95.5 [28], 95 [71], and 94.5% [72], respectively. For loaded drugs, the EE of afatinib-loaded LPHNs was found to be 93.2%, while cisplatin-loaded LPHNs had EE values ranging from 20.5% to 91.8%, depending on the carrier composition and method of preparation [73,74,75]. Other drugs like curcumin [69], docetaxel [76,77], and paclitaxel [19], also showed significant EE values, with curcumin-loaded LPHNs reaching an impressive 96% EE using HPLC detection [69], while docetaxel-loaded LPHNs had EE values between 10% and 89.1%, depending on the formulation [76,77]. Additionally, other agents like 5-FU, afatinib, bevacizumab, doxorubicin, etoposide, gemcitabine, honokiol, isoliquiritigenin, methotrexate, melphalan, mitomycin C, mycophenolic acid, solasonine and solamargine, tamoxifen, thymoquinone, vorinostat, and 10-hydroxycamptothecin are loaded into LPHNs using various approaches which exhibited a varying EE%, making them promising candidates for combination therapy (Table 1).

### 3.4. Drug Release Mechanisms

The hydrophobic drugs can be carried by the LPHNs with high loading yields, and their dissolution kinetics can be controlled. A significant number of hydrophobic drugs may be directly enclosed within the hydrophobic polymer core or chemically bonded to the polymer chains in the hydrophobic polymer core. The lipid shell is predicted to improve drug loading yield by preventing tiny drug molecules from readily diffusing out of the polymer core and reducing the rate of water entering the polymer core, which delays the breakdown of the polymer and the release of the drug from the LPHN [99].

Drug diffusion and polymer erosion are generally employed to release drugs from the LPHNs. The hydrolysis of the linkers between the drug and polymer chains and subsequent drug diffusion regulates the release of drug molecules. The degradation rate and particle size affect the drug release profile from the hybrid nanoparticles [100]. In addition, a remote radio frequency magnetic field is also used for drug release from the LPHN system. For instance, the magnetic field-activated LPHN nanosystem loaded with camptothecin showed long-term stability in terms of particle size and polydispersity index in phosphate-buffered saline, and stimuli-responsive drug release [101]. Typically, drug release studies are carried out using the dialysis technique. To put it briefly, a large volume release medium at 37 °C with moderate agitation is placed in a dialysis cassette carrying drug-loaded nanoparticles. The drug molecules will frequently leak into the release media from the nanoparticles. For quantification, the drug molecules which have been released or which remain inside the nanoparticles are collected at various time points using analytical methods including high-performance liquid chromatography (HPLC) and mass spectrometry [102,103].

### 3.5. Oral Bioavailability Enhancement (Preclinical Studies)

LPHNs can enhance the oral bioavailability of anti-cancer drugs via several mechanisms. LPHNs protect the encapsulated drug from degradation in harsh gastrointestinal environments, such as low pH and enzymatic degradation [60,104]. Paclitaxel-loaded LPHNs showed an absolute bioavailability of 21.95% compared with that of paclitaxel conventional formulation, which had an oral bioavailability of 4.75%. Cytochrome 450 and P-glycoprotein (P-gp) inhibitors incorporation further improved the oral bioavailability of paclitaxel to 42.60%, and bioavailability almost increased almost eight-fold [105]. LPHNs can also increase the solubility of many anti-cancer hydrophobic drugs. Subsequently, it leads to their improved absorption and oral bioavailability [104]. Another special characteristic of LPHN formulation is to target specific cancer cells by modifying the surface of the nanoparticles with ligands such as folic acid, and folate, which bind to specific receptors on the cancer cell surface [19,57]. Subsequently, bioadhesive systems can extend the retention time of the insoluble drugs, causing it to remain for a longer period of time in the GI tract, so they are frequently used to improve oral drug absorption. The interaction of nanoparticles with mucin improves mucus penetration and enhances bioavailability [106]. Mucin is the main component of mucus, which is secreted by the mucus layer overlying the intestinal epithelium. Intestinal epithelium is considered the first line of oral absorptive barriers [107]. LPHNs of cabazitaxel showed higher mucus penetration in comparison to free cabazitaxel. The oral bioavailability of cabazitaxel was elevated from 7.7% to 56.6% (7.3 times higher) [108].

LPHNs can be designed to release the encapsulated drug in a controlled manner, providing sustained drug delivery and making it available in systemic blood circulation for a longer period of time, which results in improved bioavailability and reduces the frequency of dosing [86,109]. Tamoxifen-loaded-LPHNs exhibited enhanced oral bioavailability with a considerable increase in area under the curve (AUC) (1277.46 vs. 585.01 ng/mL·h) in comparison to tamoxifen. LPHNs showed a markedly extended half-life (27.87 25.72 vs. 17.42 12.04 h) and an enhanced mean residence duration (40.1 h) with a heightened T_max_ in comparison to pure tamoxifen. As a result, tamoxifen’s bioavailability improved by more than two-fold when incorporated into LPHNs [96].

If a drug is capable of passing through the intestinal mucus barrier, it can be absorbed in the epithelium more effectively and delivered more quickly, resulting in enhanced bioavailability [110]. Because of the potent electrostatic interactions between positively charged NPs and the negatively charged sialic groups of mucins, it has been predicted that positively charged NPs have a high affinity for mucus [111]. It was found that muco-adhesion of the positively charged paclitaxel-loaded LPHNs showed an absolute bioavailability of 21.95% compared with that of paclitaxel conventional formulation, which had an oral bioavailability of 4.75% [105].

## 4. LPHN-Based Anticancer Drug Delivery

Oral chemotherapeutic drug use has been steadily increasing. The ease of home-based therapy is the main reason for choosing anti-cancer oral therapy over intravenous therapy. The primary benefits of using oral anti-cancer drugs among oncologists include enhanced compliance among patients, drug tolerability, and ease of use. Oral chemotherapeutic drugs bioavailability variability among patients may be of greater significance than intravenous agents [112].

Various cytotoxic drug combinations, such as gemcitabine and paclitaxel tested on pancreatic cancer cell lines (XPA3), cisplatin and paclitaxel tested on ovarian cancer cell lines (A2780), and doxorubicin and camptothecin tested on breast cancer cell lines (MDA-MB-435), have been co-delivered using LPHNs as a drug delivery platform. Considering LPHN-based drug delivery, enhanced intracellular entry via endocytosis consequently overcomes their poor cellular absorption. Conjugated paclitaxel and cisplatin LPHNs demonstrated increased cytotoxicity on human ovarian cancer cells compared to free drug combinations [113].

The poor water solubility and oral bioavailability of phytochemicals like thymoquinone (THQ) restrict their use in clinical applications. The optimized LPHNs showed remarkable qualities for the best oral THQ delivery. THQ-LPHNs demonstrated exceptional gastrointestinal stability as well as storage stability under various environmental conditions. In both gastric and intestinal media, THQ-LPHNs showed nearly identical release characteristics, with an initial rapid release lasting 4 h and a subsequent sustained release lasting up to 48 h. After oral administration, THQ-LPHNs demonstrated a 4.74-fold higher bioavailability than the standard THQ solution [97].

Male Wistar rats were administered paclitaxel (10 mg/kg) orally, which resulted in a maximum plasma concentration (C_max_) of 2581 ng/mL. In contrast, paclitaxel-loaded LPHNs demonstrated a three-fold increase in drug concentration, reaching a C_max_ of 7609 ng/mL, indicating a three-fold higher bioavailability [92]. When compared to the free paclitaxel solution, Pandita et al. concluded that the drug exposure in tissues and plasma was 2-fold and 10-fold greater following oral administration of the paclitaxel-loaded LPHNs [114]. For oral delivery, paclitaxel-loaded LPHNs with a size range of 200–300 nm was developed to withstand challenging GIT conditions and increase paclitaxel’s bioavailability. Comparatively, an increase in bioavailability and elimination half-life of 1.5 and 5.5 times, respectively, was reported [115]. When compared to free paclitaxel solution, the drug exposure in plasma and tissues after the oral administration of paclitaxel LPHNs was 10 and 2 times greater, respectively [114].

The oral bioavailability of cabazitaxel was elevated from 7.7% to 56.6% (7.3 times higher) when administered through LPHNs [108]. When administered using an LPHN formulation, cisplatin exhibited a half-life (T_1/2_) of 5.4 h and a C_max_ of 17.1 L/kg/h, whereas the free drug had a far lower half-life of 1.7 h and a C_max_ of 2.3 L/kg/h. Once cisplatin was administered through LPHNs, there was an approximately three-fold increase in the half-life and a seven-fold rise in C_max_. According to an in-vivo pharmacokinetics analysis, the AUCs for cisplatin-LPHNs and free cisplatin were 247 and 16 mg/L/h, respectively [78]. Only small quantities of doxorubicin and sorafinib were able to be found after 12 h of administration, which indicates that doxorubicin and sorafinib were quickly eliminated in their free form. On the other hand, drugs loaded into LPHNs showed extended retention for up to 36 h [116]. The targeted LPHNs carrying a doxorubicin and gallic acid combo coated with hyaluronic acid showed enhanced cytotoxicity and exhibited higher distribution in tumor compared to free drug in acute myeloid leukemia bearing mice. No significant differences in drug distribution were found in the other organs [117].

Folate-modified LPHNs for targeted paclitaxel delivery showed greater tumor growth inhibition (65.78%) compared to paclitaxel-loaded LPHNs (48.38%) [19]. Paclitaxel and etoposide combinations were far more cytotoxic than either drug alone. Similar to this fact, LPHN-based administration showed much boosted cancer cell death (~45%). The therapeutic efficacy of etoposide and paclitaxel for treating osteosarcoma was improved when the drugs were combined in LPHNs [118]. Furthermore, 5-FU and sulforaphane-loaded LPHNs significantly enhanced the cytotoxicity against HCT 15 colorectal cancer cell lines. The dual-drug-loaded LPHNs demonstrated an increased apoptotic effect compared to free drugs, suggesting that the combination of 5-FU and sulforaphane within the LPHNs may synergistically improve the therapeutic efficacy, leading to more effective cancer cell killing [119]. The LPHN-based drug delivery of paclitaxel and etoposide was found to have synergistic anti-cancer effects against osteosarcoma [120]. When mice were intravenously given doses of 0.1 and 0.2 mg of doxorubicin-loaded LPHNs, there was a 70 and 100% delay in the development of the cancer, respectively. The cytotoxic activity of the LPHNs against solid tumors appeared helpful, and they increased the efficacy of treatment [121]. In addition, the cell cytotoxicity assay revealed significantly higher cell cytotoxicity of LPHNs against human breast cancer cell lines (MCF-7) compared to the free drugs mycophenolic acid and quercetin, and their combination in both concentrations was in a time-dependent manner [90]. Gemcitabine is also delivered via LPHNs, which prolongs blood circulation duration, which is beneficial for gemcitabine accumulation in breast cancer mice models. Furthermore, gemcitabine loaded with LPHNs showed improved anti-tumor activity in rats when compared to commercial product Gemko^®^ [86]. For the treatment of hormone-resistant prostate cancer, LPHNs that encapsulated tumor-targeted docetaxel increased its efficacy and safety [77].

The short half-life and mean retention time, which were responsible for the poor pharmacokinetic behavior, were compared with the high elimination rate and drug clearance of free doxorubicin and sorafinib. The pharmacokinetic characteristics of both drugs were greatly improved when they were co-loaded in LPHNs, respectively. LPHN-based drug delivery showed extended retention for 36 h [116]. Erlotinib-loaded LPHNs demonstrated a significant absorption into the cytoplasm of cells, and the in-vitro cell viability experiment demonstrated a significant decrease in the proliferation of A549 cells after 72 h [83]. Squamous cell carcinoma (SCC-7), human breast adenocarcinoma (MCF-7), and human Caucasian breast adenocarcinoma (MDA-MB-231) cells had all been more efficiently inhibited by the drug-loaded carrier when docetaxel and vorinostat were administered together through LPHNs [81].

## 5. Limitations and Prospects

LPHNs have emerged as a potential strategy for cancer therapy and drug delivery. However, there are certain limitations that need to be addressed for their successful implementation. Firstly, there are technological requirements that must be fulfilled to achieve uniformity and repeatability on a large scale. Additionally, creating LPHNs with a consistent size distribution and diverse functionalities for enhanced in-vivo performance poses challenges, particularly in traversing multiple epithelial barriers in lung tissue [122]. The limitations of LPHNs include a limited drug loading capability, which restricts the amount of drug that can be delivered per nanoparticle, potentially impacting the treatment of larger tumors or cancer tissues [77]. Stability issues, such as aggregation or degradation, can affect the ability of LPHNs to efficiently transport drugs to the desired site [123,124]. Moreover, the targeting capability of LPHNs is often limited by factors such as surface charge, size, and availability of targeting ligands [125]. Scale-up hurdles pose challenges in producing LPHNs at a sufficiently large scale for clinical usage, which may affect their accessibility and cost [126].

Despite the aforementioned limitations, there are several promising opportunities for the future of anti-cancer drug delivery using LPHNs. These opportunities include enhancing the drug-loading capacity of LPHNs through techniques like covalent bonding, prodrug conjugation, and multistage delivery systems [127]. Moreover, researchers are also developing novel polymers and protective coatings, such as polymer-coated LPHNs, to improve their stability and prolong their circulation in the bloodstream [115,128]. Advancements in targeting capabilities are being explored, including the use of tumor-specific ligands, responsive surface coatings, and biomimetic materials which allow site-specific drug release through LPHNs [129]. Modern imaging techniques, such as fluorescence labeling and magnetic resonance imaging (MRI), enable better visualization and monitoring of LPHNs within the body which facilitates precise delivery to the target site [130,131]. Additionally, LPHNs can be utilized in combination with other therapeutic approaches like gene or immune therapies, thereby augmenting the overall efficacy of cancer treatment [131]. Lastly, in order to allow widespread clinical usage of LPHNs, researchers are also developing scalable production procedures [132,133]. In overall terms, LPHNs hold an optimistic outlook for the delivery of anti-cancer drugs, and more research and development in this area might lead to the advancement of more efficient and reliable therapies for cancer.

## 6. Conclusions

LPHNs have a lot of potential as an innovative and successful drug delivery technology for anti-cancer drugs having limited oral bioavailability issues. Studies have shown that LPHNs significantly improve drug bioavailability, such as paclitaxel (21.95% compared to 4.75%), cabazitaxel (7.3-fold increase from 7.7% to 56.6%), and thymoquinone (4.74-fold increase). Tamoxifen-loaded LPHNs demonstrated more than a two-fold increase in AUC (1277.46 ng/mL·h vs. 585.01 ng/mL·h) and extended half-life. Furthermore, LPHNs have enhanced drug cytotoxicity, as seen with paclitaxel and cisplatin combinations, and improved anti-tumor activity with drugs like gemcitabine, showing a significant increase in drug exposure and therapeutic effects compared to free formulations. EE% values for LPHNs also vary across different drugs and types of lipids and polymers, highlighting their versatility. For instance, LPHNs loaded with paclitaxel achieved an impressive EE% of 85%, while gemcitabine-loaded LPHNs showed a lower EE% of 56.7%. The EE% for drugs like doxorubicin and sorafenib in LPHNs has been reported to be around 75%, while LPHNs containing docetaxel achieved EE% values as high as 87%. In contrast, lower EE% values have been observed with certain formulations, such as those loaded with camptothecin, which had an EE% of approximately 55%. The lipid DLPC and the polymer PLGA and DSPE-PEG 2000 combination provide the highest EE% of 93.2% for afatinab. Similarly, Lecithin combined with PLGA and DSPE-PEG 2000 also shows a high EE% of 96% for Curcumin.

Furthermore, LPHNs have demonstrated significant improvement in cytotoxicity, with paclitaxel and cisplatin-loaded LPHNs showing enhanced cytotoxic effects in ovarian cancer cell lines (A2780), and doxorubicin-loaded LPHNs exhibiting extended retention in the body for up to 36 h. These LPHN formulations have also been shown to increase drug exposure in tissues and plasma, which is crucial for improving cancer treatment efficacy. In conclusion, LPHNs hold a bright future in the delivery of chemotherapy drugs, and more research and development in this area may result in the creation of more effective and specialized therapies for cancer. We are sure that with additional technological and manufacturing advancements, LPHNs will eventually have a significant role in the battle against cancer, moving us one step closer to uncovering a cure against cancer.

## Figures and Tables

**Table 1:** LPHN-loaded anti-cancer drugs, composition (type of lipid, and polymer), preparation method, size, EE (%), and detection method.

Carrier System	Lipid	Polymer	Preparation Method	Size (nm)	Loaded Drug	EE (%)	Detection Method	Ref.
LPHNs	DLPC	PLGA and DSPE-PEG 2000	Single emulsification solvent evaporation	138.2	Afatinab	93.2	UV-Visible spectrophotometry	[73]
	Compritol^®^ 888 ATO, Precirol^®^ ATO 5, Miglyol^®^ 812, Lecithin and Tween^®^ 80	Poly(d,l-lactide)	Single emulsification solvent evaporation	141.2	Cisplatin	91.3	UV-Visible spectrophotometry	[78]
	DLPC	PLGA and DSPE-PEG 2000	Single emulsification solvent evaporation	138.2	Cisplatin	91.4	UV-Visible spectrophotometry	[73]
	Lipoid S75	Chitosan	Ionic gelation	258.8	Cisplatin	91.8	UV-Visible spectrophotometry	[74]
	Lecithin	PLGA and DSPE-PEG 2000	Single-step sonication	94.4	Cisplatin	20.5	UV-Visible spectrophotometry	[75]
	Lecithin, and Chol-PEG-RGD	mPEG–PLGA copolymer	W/O/W double emulsification	216.6	Curcumin	96	HPLC	[69]
	Soybean Lecithin	PLGA, DSPE-PEG (2000), and DSPE-PEG2000-RGD	W/O/W emulsion solvent evaporation	110	Docetaxel	77.6	HPLC	[79]
	Compritol, Soybean Lecithin,	PEI and PEG	Single-step solvent evaporation	216.3	Docetaxel	89.1	HPLC	[76]
	Phosphatidylcholine, Cholesterol, and Sphingosine FTY720	PLGA and 18:0 PEG2000 PE	Single-step solvent evaporation	141.5	Docetaxel	10	LC-MS/MS	[77]
	Lecithin	PLGA, and PEG	Single-step nanoprecipitation	19	Docetaxel	59	HPLC and UV-Visible spectrophotometry	[80]
	Capryol 90 (Capryol), DDAB, and TPGS	PEG-b-PAsp (Poly(ethylene glycol)-block-poly(aspartic acid))	Double-step solvent evaporation	232.4	Docetaxel and vorinostat (combo)	75.8	HPLC	[81]
	Stearic Acid, and Oleic Acid	Eudragit, Ethyl Cellulose, and Sodium Lauryl Sulfate	Dual-step sonication	185.4	Doxorubicin	95.5	UV-Visible spectrophotometry	[28]
	Epoxidized Soybean Oil, and Stearic Acid	Pluronic F68, and	Two-step solvent evaporation	80–350	Doxorubicin	60-80	UV-Visible spectrophotometry	[82]
	Hydrogenated soy phosphatidylcholine, and DSPE-PEG2000	Polycaprolactone (10 kDa, 42 kDa, and 80 kDa)	Single-step sonication	150–180	Erlotinib	67	HPLC	[83]
	Soy phosphatidylcholine	Amine-PEG-Aldehyde (CHO-PEG-NH2, MW 2 kDa)	Single-step sonication	100−120	Etoposide and bevacizumab (combo)	85.3	UV-Visible spectrophotometry	[84]
	Illipe Butter, Lecithin, Phosphatidylcholine, and Cholesterol	PLGA, PEG, Eudragit, Ethyl Cellulose, and Chitosan	Coaxial electrospraying	104.1	5-FU and Reglan (combo)	91	UV-Visible spectrophotometry	[85]
	Soya phosphatidylcholine, and DSPE-PEG2000	PLGA 50:50 (MW: 38–54 kDa), PLGA 65:35 (MW: 24–38 kDa), and PVA (MW 31–50 kDa, 87–89% hydrolyzed)	Two-step solvent evaporation	~200	Gemicitabine	45	HPLC	[86]
	Soybean Lecithin, and Cholestrol	mPEG-PLGA (75:25), and ε-Polylysine	Two-step solvent evaporation	60	Gemicitabine and HIF-1 alpha SiRNA (combo)	42	HPLC	[17]
	Soybean phosphatidylcholine, and DSPE-PEG2000	Poly(β-amino ester)	Single-step solvent evaporation	105.3	Honokiol	75	HPLC	[87]
	Lecithin, and DSPE-PEG-Mal	PLGA	Single-step nanoprecipitation	137.2	Isoliquiritigenin	91	HPLC	[88]
	Soy phosphatidylcholine, Cholesterol, and Tween 80	Chitosan, and PLGA	Single-step sonication	154	Methotrexate and conferone (combo)	85.1, and 78.4	UV-Visible spectrophotometry	[89]
	Soya lecithin	PVA, and PLGA ((50:50 molar ratio)	Single-step nanoprecipitation	162.8	Melphalan	94.5	UV-Visible spectrophotometry	[72]
	Soybean phosphatidylcholine, and DPPE	Poly(d,l-lactide) (10 kDa)	Single-step solvent evaporation	215.6	Mitomycin C	95	UV-Visible spectrophotometry	[71]
	Pluronic^®^ F-68, Soya lecithin, and 1,2-distearoyl-sn-glycero-3-phosphoethanolamine-N-[amino(polyethylene glycol)]	PLGA	Single-step nanoprecipitation	136 and 176	Mycophenolic acid and quercetin	78.2	HPLC	[90]
	Lecithin, DSPE-PEG-2000, DSPE-PEG-3400-Mal and Cholesterol	mPEG-PLA (Diblock copolymer)	W/O/W double emulsion solvent evaporation	160	5-FU, irinotecan, oxaliplatin	96	HPLC	[70]
	Soybean Lecithin	PLGA, PVA, and CMC	W/O/W double emulsion solvent evaporation	176	Paclitaxel	92	UV-Visible spectrophotometry	[91]
	Stearyl amine, and Soya lecithin	PLGA	Single step nanoprecipitation	207	Paclitaxel	67.5	HPLC	[92]
	DSPE, DSPE-PEG-2000,and Soybean lecithin (90–95% phosphatidylcholine)	PLGA (50:50 monomer ratio)	Nanoprecipitation technique combined with self-assembly	-	Paclitaxel	81.3	HPLC	[93]
	Polyvinyl alcohol	PLGA	Single-step nanoprecipitation	305.4	Paclitaxel	62.6	HPLC	[94]
	DSPE-PEG2000	Poly(ε-caprolactone), PEG, and PCL-PEG-PCL (amphiphilic copolymer)	Single-step solvent evaporation	279.9	Paclitaxel	91.1	HPLC	[19]
	Illipe Butter	Chitosan	Single-step solvent evaporation	130	Solasonine and solamargine (combo)	91.0	HPLC	[95]
	Lecithin (70% phosphatidylcholine), and Poloxamer 407	Low molecular weight chitosan	Single step emulsification solvent evaporation	169.6	Tamoxifen	72.1	HPLC	[96]
	Phospholipon 90G (PL-90G)	Chitosan (CHS; 85% deacetylated), and Poloxamer-188 (P-188)	Single-step nanoprecipitation	179.6	Thymoquinone	85.4	HPLC	[97]
	Capryol 90, TPGS, and DDAB	PEG-b-PAsp	Single emulsification-solvent evaporation	232.4	Vorinostat and docetaxel (combo)	73.7 and 75.8	HPLC	[81]
	Egg lecithin, DSPE, and DSPE-PEG	PLGA (50:50, Mw 50,000)	Single emulsification-solvent evaporation	220.4	10-hydroxycamptothecin	72.6	UV-Visible spectrophotometry	[98]

Abbreviations: 1,2-Dilauroyl-sn-glycero-3-phosphocholine (DLPC), 1,2-Distearoyl-sn-glycero-3-phosphoethanolamine (DSPE), 1,2-Distearoyl-sn-glycero-3-phosphoethanolamine-N-[amino(polyethylene glycol)-2000] (DSPE-PEG), 1,2-Distearoyl-sn-glycero-3-phosphoethanolamine-N-[methoxy(polyethylene glycol)-2000] (18:0 PEG2000 PE), Cholesterol conjugated with PEG and Arginine-Glycine-Aspartic acid (RGD) (Chol-PEG-RGD), Didodecyldimethylammonium bromide (DDAB), D-α-Tocopherol Polyethylene Glycol 1000 Succinate (TPGS), Hypoxia Inducible Factor (HIF), Polyethylene glycol and poly(DL-lactide-co-glycolide) (mPEG–PLGA copolymer), Monomethoxy poly(ethylene glycol)-poly(lactic-co-glycolic acid) (mPEG-PLGA), Polyethylene glycol-block- Poly(aspartic acid) (PEG-b-PAsp),
